# H9N2 Avian Influenza Virus Protein PB1 Enhances the Immune Responses of Bone Marrow-Derived Dendritic Cells by Down-Regulating miR375

**DOI:** 10.3389/fmicb.2017.00287

**Published:** 2017-03-22

**Authors:** Jian Lin, Jing Xia, Chong Z. Tu, Ke Y. Zhang, Yan Zeng, Qian Yang

**Affiliations:** ^1^Department of Zoology, College of Life Science, Nanjing Agricultural UniversityJiangsu, China; ^2^Department of Histoembryology, College of Veterinary Medicine, Nanjing Agricultural UniversityJiangsu, China

**Keywords:** H9N2 AIV, PB1, miRNA, dendritic cells, immune regulation

## Abstract

Polymerase basic protein 1 (PB1), the catalytic core of the influenza A virus RNA polymerase complex, is essential for viral transcription and replication. Dendritic cells (DCs) possess important antigen presenting ability and a crucial role in recognizing and clearing virus. MicroRNA (miRNA) influence the development of DCs and their ability to present antigens as well as the ability of avian influenza virus (AIV) to infect host cells and replicate. Here, we studied the molecular mechanism underlying the miRNA-mediated regulation of immune function in mouse DCs. We first screened for and verified the induction of miRNAs in DCs after PB1 transfection. Results showed that the viral protein PB1 down-regulated the expression of miR375, miR146, miR339, and miR679 in DCs, consistent with the results of H9N2 virus treatment; however, the expression of miR222 and miR499, also reduced in the presence of PB1, was in contrast to the results of H9N2 virus treatment. Our results suggest that PB1 enhanced the ability of DCs to present antigens, activate lymphocytes, and secrete cytokines, while miR375 over-expression repressed activation of DC maturation. Nevertheless, PB1 could not promote DC maturation once miR375 was inhibited. Finally, we revealed that PB1 inhibited the P-Jnk/Jnk signaling pathway, but activated the p-Erk/Erk signaling pathway. While inhibition of miR375 -activated the p-Erk/Erk and p-p38/p38 signaling pathway, but repressed the P-Jnk/Jnk signaling pathway. Taken together, results of our studies shed new light on the roles and mechanisms of PB1 and miR375 in regulating DC function and suggest new strategies for combating AIV.

## Introduction

The influenza virus contains eight segments of a single-stranded RNA genome with negative polarity. The H9N2 subtype avian influenza virus (AIV), classified as a low pathogenic AIV, has high genetic variability and has shown both increases in virulence and ability to cross the host barrier (Peiris et al., 1999; Jin et al., 2014; Shaib et al., 2014; Zhou et al., 2014). Since the H7N9 and H10N8 AIV outbreaks in 2013 resulted from recombination between H9N2 and other influenza subtypes, H9N2 AIV is a subject of intense research (Fang et al., 2013; Pu et al., 2015). The virus polymerase complex of H9N2 AIV, consisting of the polymerase basic protein 1 (PB1), polymerase basic protein 2 (PB2) and polymerase acidic protein (PA) subunits, has been reported to charge for catalyzing both viral RNA genome replication and transcription. Previous studies have shown that PB1 serves as a core subunit to incorporate PA and PB2 into the polymerase complex by directly interacting with PA and PB2 (Hemerka et al., 2009). Recently studies also reveal that PB1 interacted with PA was an attractive target for drug treatment (Massari et al., 2016; Swale et al., 2016).

There is a continuing need for novel anti-influenza therapeutics using new targets or creative strategies and the pathogenicity of a virus is determined not only by its characteristics but also by the host immune response (O'Donnell and Subbarao, 2011). Dendritic cells (DCs) is a professional and effective antigen-presenting cells in the innate immune response (Tucci et al., 2014). Influenza virus is a human pathogen and also naturally infects a large range of animals. Previous studies have found that the mouse model of influenza virus infection is useful for understanding host immune responses and host-pathogen interaction. Zhou found that miR2911, a honeysuckle (HS)-encoded atypical microRNA (miRNA), directly targets Influenza A virus with various subtypes (Zhou et al., 2015). Also, Isakova-Sivak and colleagues have used the mouse model to study the infectivity, immunogenicity and cross-protective efficacy of live attenuated influenza vaccines containing nucleoprotein from cold-adapted or wild-type influenza virus (Isakova-Sivak et al., 2017). Consequently, because human blood and humanized mice were not available for our studies, we developed a suitable animal model to study AIV responses in mammalian DCs. AIV infection affects the maturation, antigen presenting ability, and cytokine secretion of DCs (Lin et al., 2014). The binding of pathogen-associated molecular patterns to receptors expressed by DCs may activate DCs (Lopez et al., 2004; Liang et al., 2013), but it remains unclear how AIVs produce changes in DCs and how DCs respond to AIV infection.

MicroRNA (miRNA) have emerged as key regulators of innate immunity and modulate the ability of DCs to present antigens and secrete cytokines (Gantier et al., 2007; Cheng et al., 2012; Smyth et al., 2015). For example, miR-24 has been shown to be regulated during macrophage and dendritic cell differentiation potentiates innate immunity (Fordham et al., 2015). MicroRNA-146a reported to regulate human dendritic cell apoptosis and cytokine production by targeting TRAF6 and IRAK1 proteins (Park et al., 2015). Furthermore, AIV infection leads to the differential expression of cellular miRNA in chickens and mice, and miR491 and miR654 inhibit the replication of H1N1 virus through binding to PB1 in MDCK cells (Song et al., 2010). Since miRNA have the ability to modulate DC function, and our previous research work demonstrated that H9N2 AIV significantly influenced miRNA expression of DCs, the purpose of our study was to investigate the role miRNA play in regulating the immune response of DCs to PB1 stimulation.

## Results

### miRNA expression following H9N2 AIV infection and viral fragment transfection

To study how H9N2 might control miRNA expression, three segments of H9N2 AIV (PB1, PA, and NP) were cloned into pcDNA3.1 and transfected into bone marrow-derived DCs (BMDCs) (Supplementary Image 1). The expression of selected miRNAs was then examined by reverse transcription quantitative real-time PCR (RT-qPCR). Interestingly, for all of the miRNAs up-regulated by H9N2, PB1, PA, and NP significantly repressed their expression. For the miRNAs down-regulated by H9N2, the PB1 segment also mostly reduced their expression, especially for miR339, miR375, and miR146 (Figures 2A,B). Segments PB1, PA, and NP are involved in the transcription and replication of the AIV RNA genome (Bouvier and Palese, 2008).

**Figure 1:**
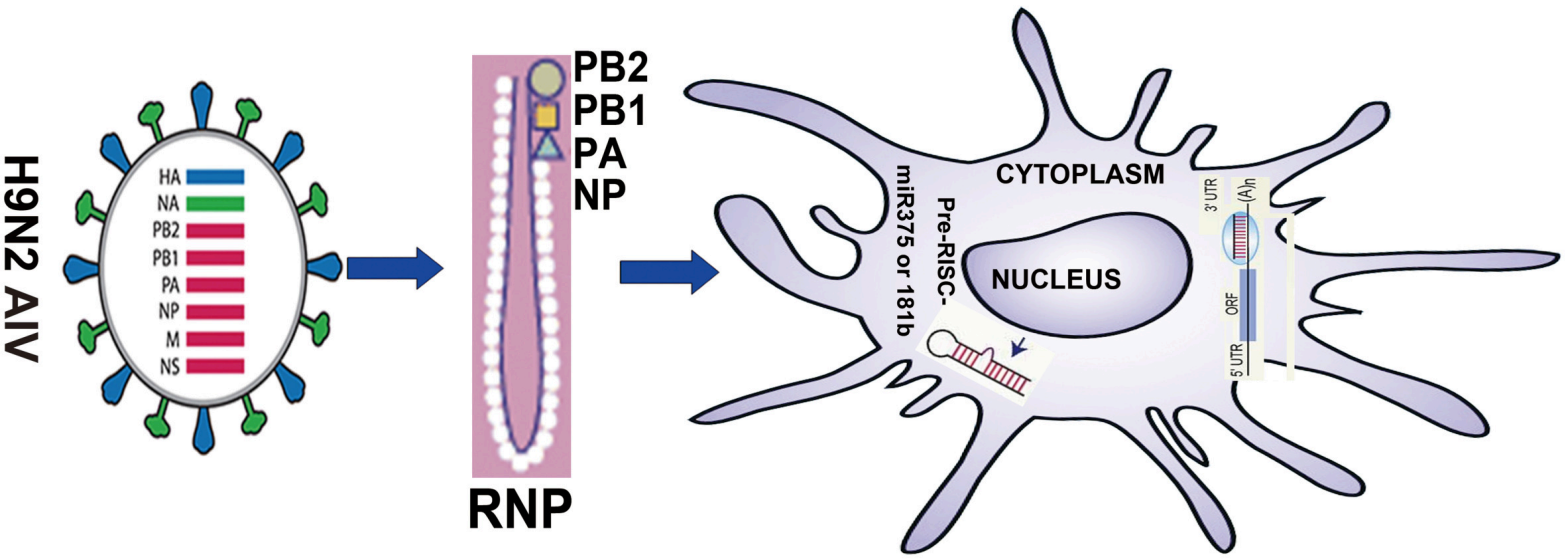
**Summary of the research strategy**.

**Figure 2:**
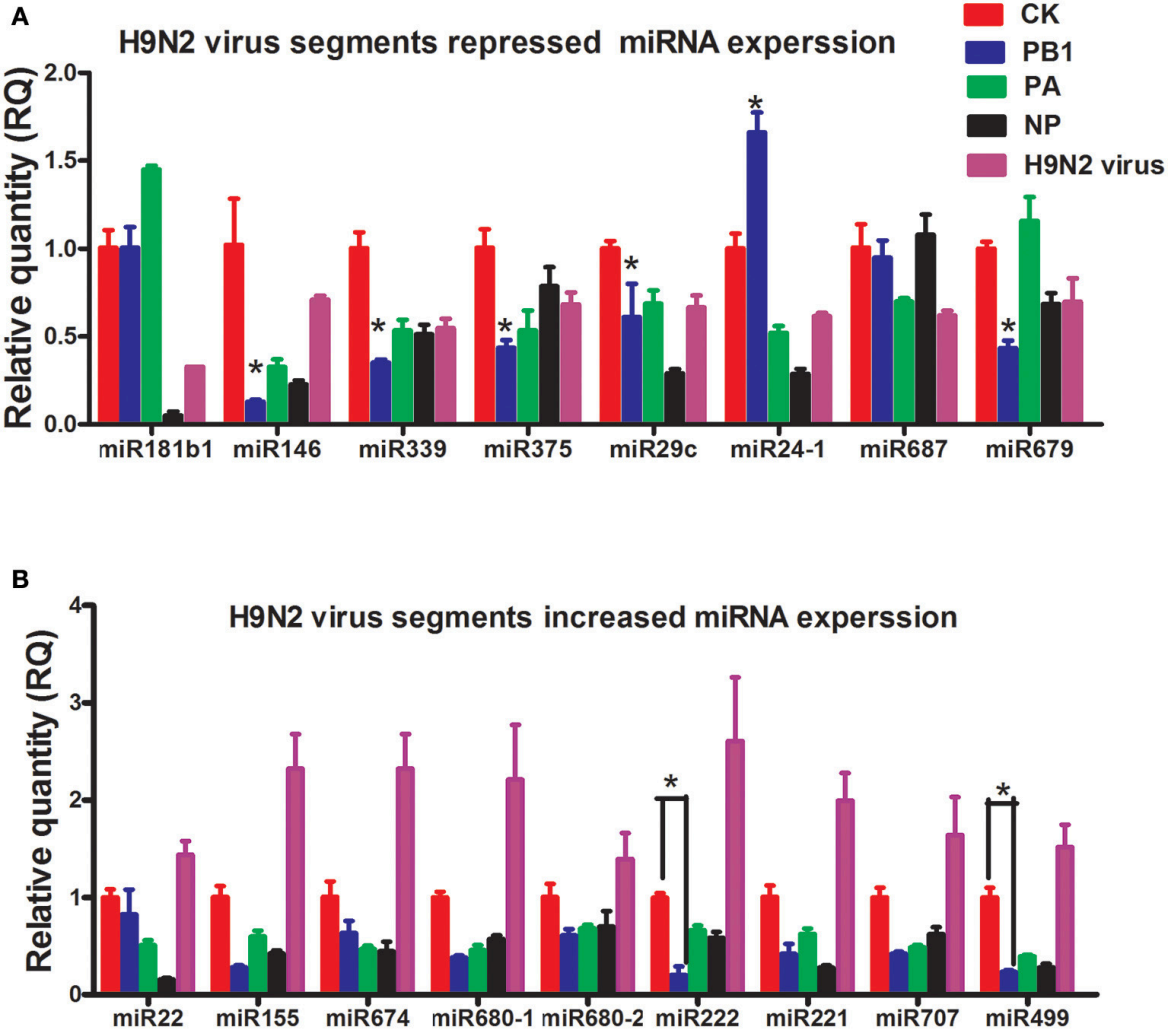
**Results of the qPCR analysis of select miRNAs following stimulation by PB1, PA, or NP. (A)** The expression levels of down-regulated miRNAs stimulated by PB1, PA, or NP (^*^P < 0.01, the significance of the data was determined by one-way ANOVA with Duncan test) (CK: blank DCs; PB1, PA, and NP: plasmid over-expressed DCs; LPS: 1 μg/ml, each times, three 4–6 week wild-type male C57BL/6 mice were sacrificed to isolated BMDCs and experiments were performed at least in triplicate). **(B)** The expression levels of up-regulated miRNAs stimulated by PB1, PA, or NP (^*^P < 0.01, the significance of the data was determined by one-way ANOVA with Duncan test) (CK: blank DCs; PB1, PA, and NP: plasmid over-expressed DCs; LPS: 1 μg/ml).

### Activation of mouse BMDCs by PB1

**Figure 3:**
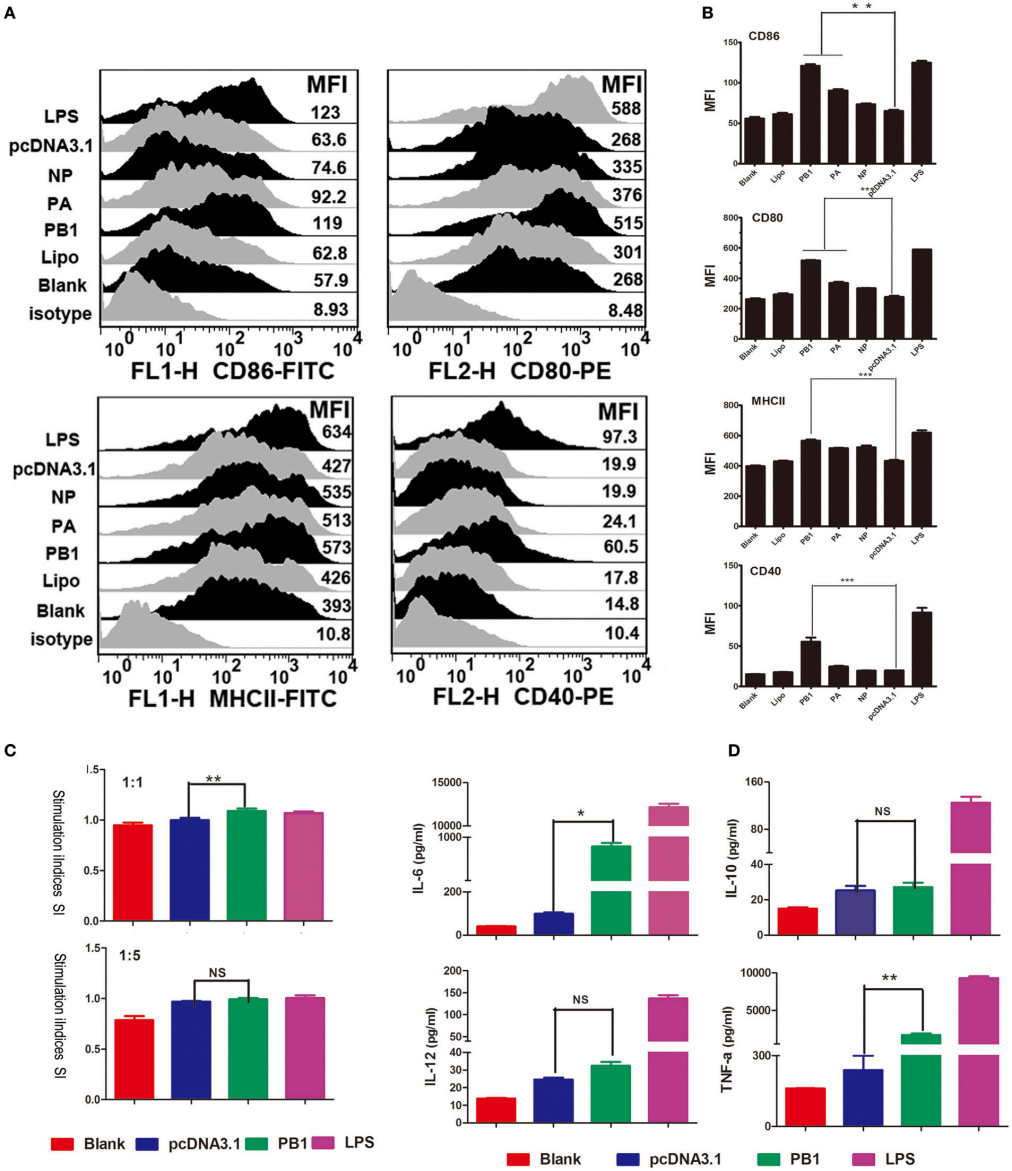
**Immune activation of BMDCs stimulated by PB1. (A)** Flow cytometric analysis of the phenotypic alterations in DCs stimulated with PB1, PA, or NP (i.e., the expressions of CD40, CD80/86, and MHCII on BMDCs stimulated with PB1, PA, or NP). (Isotype: IgG2a for CD40, IgG1 for CD80, and CD86, IgG2b for MHCII; Blank: DCs without any treatment; Lipo: DCs added the same lipofectame as transfection groups; PB1, PA, and NP: plasmids transfected DCs; pcDNA3.1 group: plasmid pcDNA3.1 transfected DCs; LPS: Positive control, 1 μg/ml LPS, three 4–6 week wild-type male C57BL/6 mice were sacrificed to isolated BMDCs and experiments were performed at least in triplicate). **(B)** The MFI of CD40, CD80/86, and MHCII (^*^P < 0.05, ^**^P < 0.01, or ^***^P < 0.001 the significance of the data was determined by one-way ANOVA with Tukey's multiple comparison test). **(C)** PB1-stimulated BMDCs stimulated the proliferation of naive T cells in mixed-lymphocyte reactions (MLR). The stimulator cells were BMDCs stimulated with or without PB1, pcDNA3.1, or LPS at 37°C for 24 h. All experiments were performed at least in triplicate. Significant differences between the treated and pcDNA3.1 groups are expressed as ^*^P < 0.05 or ^**^P < 0.01. The significance of the data was determined by one-way ANOVA with Tukey's multiple comparison test. **(D)** Cytokine release from PB1-stimulated BMDCs was measured by enzyme-linked immunosorbent assays (ELISAs). Data for IL-6, IL-10, IL-12, and TNF-α are shown as means ± standard deviation (SD) of three samples. Significant differences between the treated and pcDNA3.1 groups are expressed as ^*^P < 0.05 or ^**^P < 0.01. The significance of the data was determined by one-way ANOVA with Tukey's multiple comparison test.

We first examined the phenotypic changes in BMDCs transfected with PB1, PA, and NP. The transfection efficiency of PB1, PA, and NP was detected by RT-qPCR, and results are listed in Supplementary Data Sheet 1. Fluorescence-activated cell sorting (FACS) suggested that the mean fluorescence intensity (MFI) of MHCII was significantly enhanced by PB1, as were the co-stimulatory molecules CD40, CD80, and CD86 (Figures 3A,B). PA segment up-regulated CD80 and CD86, whilst NP had no effect on them (Figures 3A,B). Next, we assessed the ability of DCs to activate T lymphocytes and secrete cytokines. As shown in Figures 3C,D, PB1-stimulated DCs showed enhanced stimulation at a ratio of 1:1, and they expressed higher levels of interleukin-6 (IL-6) and tumor necrosis factor-α (TNF-α) than did the pcDNA3.1-transfected controls (*P* < 0.05). Whilst the LPS stimulation (positive control) were demonstrated to enhance the ability of DCs to present antigens, activate T lymphocytes, and secrete cytokines.

### The immune function of miR375 and miR181b in regulating mice BMDCs

**Figure 4:**
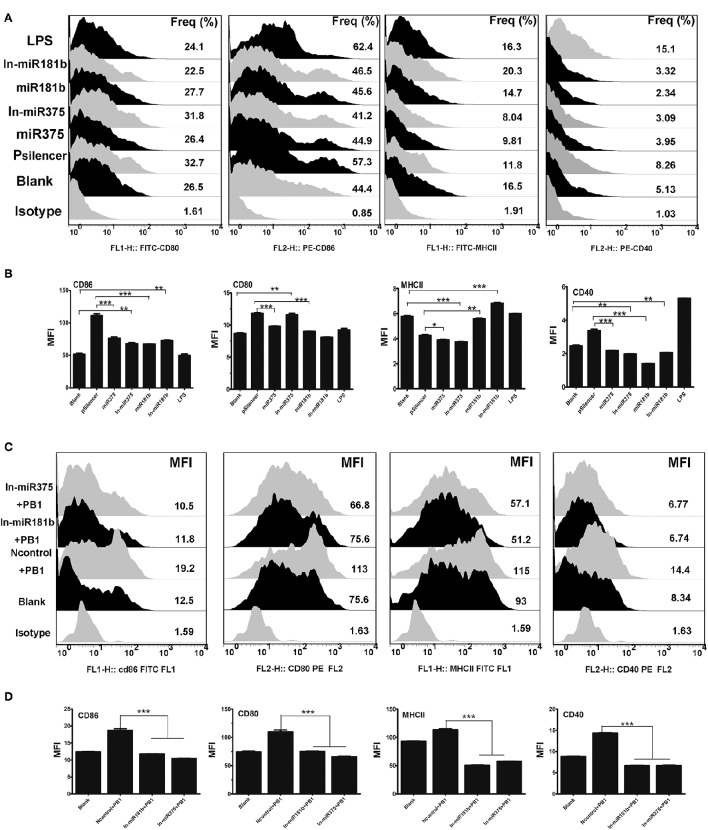
**The immune function of BMDCs stimulated by miR375 andmiR181b. (A)** Flow cytometric analysis of the phenotypic alterations in DCs stimulated by miR375, miR181b, In-miR375, and In-miR181b (i.e., the expressions of CD40, CD80/86, and MHCII on BMDCs stimulated by miRNAs). (Isotype: IgG2a for CD40, IgG1 for CD80 and CD86, IgG2b for MHCII; Positive control, 1 μg/ml LPS, three 4–6 week wild-type male C57BL/6 mice were sacrificed to isolated BMDCs and experiments were performed at least in triplicate). **(B)** The MFI of CD40, CD80/86, and MHCII. (^*^P < 0.05, ^**^P < 0.01, or ^***^P < 0.001 the significance of the data was determined by one-way ANOVA with Tukey's multiple comparison test). **(C)** PB1 mediated BMDCs activation when miR375 and miR181b were inhibited (100 μg/ml inhibitor for each miRNA, Isotype: IgG2a for CD40, IgG1 for CD80 and CD86, IgG2b for MHCII, three 4–6 week wild-type male C57BL/6 mice were sacrificed to isolated BMDCs and experiments were performed at least in triplicate). **(D)** The MFI data for CD40, CD80/86, and MHCII. (^*^P < 0.05, ^**^P < 0.01, or ^***^P < 0.001 the significance of the data was determined by one-way ANOVA with Tukey's multiple comparison test).

Recent studies have shown that miRNA regulate the immune responses of BMDCs (Wu et al., 2012). As H9N2 and PB1 significantly down-regulated the expression of miR375 (Figure 2), we examined the functions of miR375 in BMDCs. MiRNA over-expression vectors were constructed and validated by digesting with restriction enzyme Hind III and BamHI (Supplementary Image 2). FACS showed that miR375 over-expression decreased the percentage of CD80-, CD86-, CD40-, and MHCII when compared with the pSilencer4.1 group. While inhibition of miR375 greatly down-regulated the MFI of CD80 and CD86, but up-regulated the MFI of CD40 and MHCII when compared with the blank group (Figures 4A,B). Moreover, results also shown that the abundance of miR181b significant down-regulated all the surface -markers except MHC-II.). Interestingly, we found that the inhibition of miR181b significant increased the MFI of MHCII and CD-86 when compared with the blank group (Figures 4A,B).

### Inhibition of endogenous miR375 and miR181b blocked PB1-induced phenotypic alterations in BMDCs

Previous studies demonstrated that PB1 and a number of miRNAs, including miR375 and miR181b, can influence the phenotype of BMDCs. Thus, we investigated whether the PB1-induced changes in DCs are mediated by miRNAs. To test this hypothesis, miRNA inhibitors were added to DCs to repress endogenous miRNAs before PB1 transfection. FACS revealed that the inhibition of endogenous miR375 and miR181b decreased the expression of co-stimulatory molecules (CD80/CD86 and CD40) and MHCII, which was induced by PB1 (*P* < 0.05; Figures 4C,D).

### Effects on signaling pathways stimulated by PB1, miR375 and miR181b

**Figure 5:**
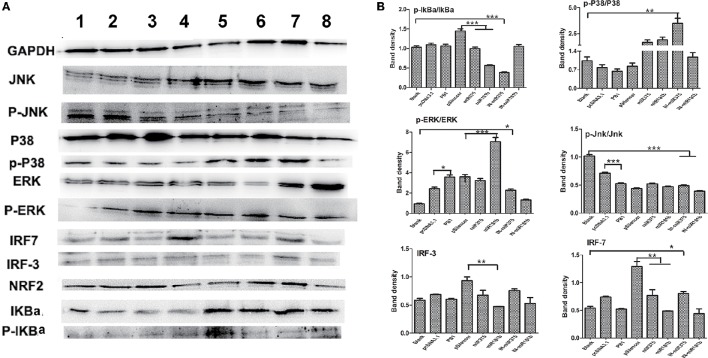
**Regulatory protein expression on BMDCs stimulated by PB1 and miRNAs as determined by Western blotting. (A)** Western blot results for IkBa, P38, ERK, Jnk, IRF-3, and IRF-7 in cells stimulated by PB1, miR375 and miR181b (lane 1: blank group; lane 2: pcDNA3.1 stimulated group; lane 3: PB1 stimulated group; lane 4: pSilencer4.1 stimulated group; lane 5: miR375 stimulated group; lane 6: miR181b1 stimulated group; lane 7: InmiR375 stimulated group; lane 8: In-miR181b1 stimulated group; three 4–6 week wild-type male C57BL/6 mice were sacrificed to isolated BMDCs and experiments were performed at least in triplicate). **(B)** The expression levels of IkBa, P38, ERK, Jnk, IRF-3, and IRF-7 in cells stimulated by PB1, miR375 and miR181b (the data shown are the means ± standard error of the mean of double wells from three independent experiments). The levels of significance are identified by ^*^P < 0.05, ^**^P < 0.01, or ^***^P < 0.001.

Mitogen-activated protein kinase (MAPK) pathways exist in all eukaryotes and control a wide range of cellular processes, such as proliferation, differentiation, and survival (Kolch, 2000). We previously demonstrated that H9N2 AIV activates interferon (IFN) regulatory factor (IRF)-7. In this study, we first found that PB1 not only significant activated the p-Erk/Erk signaling pathway, but also inhibited the P-Jnk/Jnk signaling pathway when compared with the pcDNA3,1 group. Then, we found that over expressed miR375 significant decreased the expression level of p-IkBa/IkBa and IRF7, whilst the over expression of miR181b significant decreased the expression level of p-IkBa/IkBa, IRF3 and IRF7, but significant increased the expression level of p-Erk/Erk. Furthermore, we found that the inhibition of miR375 hugely up-regulated the expression level of p-p38/p38, p-Erk/Erk and IRF7, but down-regulated the expression level of p-IkBa/IkBa and p-Jnk/Jnk which suggested activation of P38, Erk, NF-κB, and IFN signaling pathway. Finally, the inhibition of miR181b significant repressed the p-Jnk/Jnk signaling pathway when compared with the blank group (Figures 5A,B).

## Discussion

The interactions between miRNA and DCs are important for AIV infection. In this report, we focused on miR375, which exhibited decreased expression in the PB1 segment stimulated group. DCs play an important role in the generation and maintenance of immune responses (Smyth et al., 2015). There are three standards for evaluating the immune function of BMDCs, including phenotypic alterations, the ability to activate T lymphocytes and the ability to secrete cytokines (Banchereau et al., 2000). Our study suggests that the immune function of DCs, including phenotypic alteration, T lymphocyte activation, and cytokine secretion, was greatly stimulated by PB1. Also, we demonstrated that expression of miR375 was suppressed by PB1.

MiRNAs repress key regulatory components of the innate immune response and markedly affected the capacity of DCs to present antigens and secrete cytokines. MicroRNA-375 was observed to influence cell proliferation, apoptosis and differentiation through the Notch signaling pathway, while microRNA-181b modulated the secretion of TNF-α and IL-1β in macrophages (Zhang et al., 2015; Wang et al., 2016). Our research suggests that increased expression of miR375 attenuated the DC immune responses induced by PB1. PB1 over-expression increased the levels of CD80-, CD86-, MHCII-, and CD40 in cultured BMDCs, whilst the inhibition of endogenous miR375 had the opposite effect (Figures 4C,D). MiR375 also modestly decreased the expression of CD80; this effect was reversed by inhibiting endogenous miR375. Also, miR181b repressed the expression of CD40 and MHCII; this inhibition effect was relieved when endogenous miR181b was silenced. All four surface markers are characteristics of fully mature DCs and represent different functionalities (Geissmann et al., 2010). The major histocompatibility complex class II (MHCII) are family of molecules normally found on antigen-presenting cells such as DCs and mononuclear phagocytes. The MHCII-dependent pathway of antigen presentation is called the exogenous pathway, which has been shown to be regulated by miRNAs (Tomasi et al., 2010). Over-expression of PB1 enhances the ability of DCs to express the surface markers, activate lymphocytes and secrete inflammatory cytokines. Whilst the addition of miR181b decreased the expression of MHCII and CD40, this effect can be repressed by inhibiting expression of miR181b. Thus, miR181b may enhance the function of DC by down-regulating surface maturation molecules MHCII. PB1 increased the expression of the pro-inflammatory cytokines IL-6 and TNF-α, but had no effect on the anti-inflammatory cytokines IL-10 and IL-12, which seem to trigger Th17 programming in the BMDC. DCs also promote Th1 responses via IL-12 (de Jong et al., 2002).

IFN-α, controlled primarily by IFN regulatory factor 3 (IRF-3) and IFN regulatory factor (IRF-7), plays a crucial role in host defense processes against viral infection (Taniguchi and Takaoka, 2002; Yanai and Taniguchi, 2008). H9N2 AIV infection could result in the activation of IRF-7 on DCs (Lin et al., 2014). Our results show that IRF-3 and IRF-7 were all down-regulated in miR375 and miR181b groups, while inhibition of endogenous miR375 and miR181b significantly decreases IRF-3 and IRF7, suggesting that miR375 and miR181b are necessary for the production of IFN-α.

In summary, our results suggest that on the one hand, H9N2 virus protein PB1 can enhance the ability of DC to induce their phenotype, activate lymphocytes and secrete cytokines, and this effect may be accomplished by reducing the Jnk signaling pathway and activating the Erk signaling pathway. On the other hand, our results also suggest that miR375 can inhibit maturation of DC by decreasing expression of surface markers. Here, we demonstrated a previously unidentified role for PB1 in the regulation of murine immune responses of DCs, which was mediated by miR375 and miR181b. We propose that PB1 may enhance the function of DC by down-regulating miR375. Thus, miR375 may have a previously uncharacterised immunomodulatory role that can activate DCs for defense against H9N2 AIV (Figure 1).

## Methods

### Ethics statement

SPF C57BL/6 and BALB/c mice were obtained from Comparative Medical Center of Yang Zhou University. This study was approved by the Ethical Committee of Animal Experiments of the College of Veterinary Medicine, Nanjing Agricultural University. All animal care and use were conducted in strict accordance with the Animal Research Committee guidelines of the College of Veterinary Medicine, Nanjing Agricultural University.

### Plasmids and cell culture

Three RNA segments that encode proteins involved in viral replication (PB1, PA, and NP) were amplified from the H9N2 virus and cloned into pcDNA3.1 (Invitrogen). MiRNAs (miR-375 and miR-181) were amplified and cloned into pSilencer4.1 (Invitrogen). Bone marrow-derived dendritic cells (BMDCs) were prepared from the femurs and tibias of sacrificed 4–6 week wild-type male C57BL/6 mice and treated with red blood cell lysis buffer (Beyotime) (Lin et al., 2014). Briefly, bone marrow cells were flushed from the tibias and femurs and cultured in complete medium (RPMI1640 (Invitrogen) with 10% FBS (Hyclone), 1% streptomycin and penicillin, 10 ng/ml recombinant granulocyte-macrophage colony-stimulating factor (GM-CSF) and IL-4 (Peprotech) and plated in 6-well plates. At day 6, the non-adherent, relatively immature DCs (1 × 10^6^ cells/ml) were harvested and centrifuged to remove debris and dead cells, then cultured overnight in complete medium and transfected with different plasmids for subsequent assays. Transferred cell samples (1 × 10^6^ cells) were washed twice with PBS and incubated at 4°C for 30 min with the following monoclonal antibodies (anti-mouse CD11c (N418), anti-mouse CD40 (1C10), anti-mouse CD86 (GL1), anti-mouse MHC (major histocompatibility complex) class II (M5/114.15.2) and anti-mouse CD80 antibody (16-10A1), respectively (eBioscience). Finally, cells were analyzed using a Fluorescence Active Cell Sorter (FACS) (BD, FACS Aria) after two separate washes.

### The choice of miRNAs and quantitative PCR validation

To test which viral protein had the largest effect on expression of miRNAs, we amplified three replication related RNA segments (PB1, PA, and NP) and then cloned these into pcDNA3.1 vector. Primers are listed in Supplementary Table 1. Based on the microarray result, 9 up-regulated and 8 down-regulated genes were selected for RT-qPCR verification. Small RNAs were purified using the miRNeasy mini kit (Qiagen) and reverse transcribed to cDNA by miScript Reverse Transcriptase. QuantiTect SYBR Green PCR master mix (Qiagen) was used to perform qPCR according to the manufacturer's instructions. miRNA expression was normalized to the internal control 5S rRNA. Primers for 17 selected miRNAs are listed in Supplementary Table 2. All assays were performed in triplicate. Relative expression levels were calculated using the 2-ΔΔCt method (Livak and Schmittgen, 2001).

### Immune response of BMDCs stimulated by PB1

#### Surface marker alterations of BMDCs

Immature BMDCs were plated into fresh medium (1 × 10^6^ cells/ml) and transfected with one of the three vector constructs(PB1, PA, and NP), pcDNA3.1 (negative control) and LPS (1 μg/ml, positive control) for 48 h. Then cell samples (1 × 10^6^ cells, 1.5 ml tube) were collected, washed twice with PBS and incubated at 4°C for 30 min with the following monoclonal antibodies (anti-mouse CD11c, anti-mouse CD40, anti-mouse CD86, anti-mouse MHC class II and anti-mouse CD80 antibody or the respective isotype controls, respectively). After washing, cells were analyzed with a Fluorescence Activated Cell Sorter (FACS) (BD, FACS Aria)

#### Allogeneic mixed leukocyte reaction (MLR) proliferation assays

The primary T-cell stimulatory capacity of BMDCs was examined in a MLR. Untreated and variously treated BMDCs [pcDNA3.1- stimulated, PB1-stimulated and LPS-stimulated (1 μg /ml)] were used as the stimulator cells. Allogeneic lymphocytes were obtained from BALB/c mice as follows. Leukocytes were isolated from the spleens of 4 to 6 week-old allogeneic BALB/c mice with a T cell isolation kit (Miltenyi, Bergisch Gladbach, Germany) and cultured in complete RPMI 1640 medium supplementaryed with 10% FCS in 96-well plates at 37°C for 48 h. Graded numbers of responder cells (1 × 10^5^ cells/well) were added to 96-well round bottomed plates, giving responder: stimulator ratios of 1:1 or 5:1, in a culture volume of 100 μl. Cell proliferation assays was conducted with the Cell Counting Kit-8 (CCK-8, Beyotime). Each well received 20 μl CCK-8 solution and was incubated for a further 2 h at 37°C before absorbance measurement at 450 nm. All experiments were conducted in triplicate. The Stimulation Index was calculated using the formula:

$$\begin{array}{lll} \text{SI} & = & \left({\text{OD}}_{\text{sample}}-{\text{OD}}_{\text{stimulator cells only}}\right)/\\ \left({\text{OD}}_{\text{responder cells only}}-{\text{OD}}_{\text{blankcontrol}}\right). \end{array}$$

#### Cytokine analysis

Bone marrow-derived dendritic cells (BMDC) culture supernatants were collected at 24 h after treatments (Groups were divided as MLR experiments). Concentrations of TNF-α, IL-6, IL-10, and IL-12p_70_ in the supernatants were measured using the Quantikine Elisa kit (Boster) according to the manufacturer's instructions. The sensitivity of the assay was 2 pg/ml for TNF-α, and 4 pg/ml for IL-6, IL-10, and IL-12p_70_.

#### qRT-PCR validation

BMDCs was cultured and collected at 24 h after treatments with PB1, PA and NP segments. qPCR was conducted to examine expression variation for selected genes. Individual samples were diluted 1:5 and 2 μl was amplified in a 20 μl reaction containing 10 μl of SYBR Premix™ Ex Taq (TaKaRa), 0.4 μl of ROX dye II and 0.4 μM of each of the forward and reverse gene-specific primers using an ABI 7500 instrument (Applied Biosystems, USA). - We also evaluated the transcription efficiency of plasmid pcDNA3.1-PA, NP, and PB1 by qPCR. Primers are- listed in Supplementary Table 3.

### Immune response of BMDCs stimulated with miRNAs

#### Plasmid construction and phenotypic detection

To confirm that the phenotype alteration induced by PB1 is meditated by miRNA, miRNA over-expression vectors were constructed using the pSilencer4.1 vector (Invitrogen). Four selected miRNAs (miR375 and miR181b) were amplified and then cloned into pSilencer4.1. Primers are listed in Supplementary Table 4. The isolation of BMDCs and phenotypic detection were performed as described above Plasmids were transfected with lipofectame2000 reagent (Invitrogen). MiR181b and miR375 inhibitors, which were chemically modified single stranded RNAs, were designed and purchased from RiboBio to evaluate miRNA function (Guangzhou, China). Each 100 nM miRNA inhibitor (micrOFF™ mmu-miR-181b-3p inhibitor, micrOFF™ mmu-miR-375-5p inhibitor, and micrOFF™ inhibitor Negative Control) was transfected into BMDCs for 24 h to analyze their effect on DCs via detection of- phenotypic alteration with FACS.

#### miRNA inhibition experiment

To detect whether the phenotypic alteration of BMDCs induced by PB1 was mediated by miR181b or miR375, miRNAs inhibitors were transfected into BMDCs for 4 h as described above, before PB1 over-expression plasmid was transfected. After another 24 h, BMDCs were collected for phenotypic detection using FACS.

### Western blot assay

MAPK, NF-kB, and IFN-a signaling pathways control a wide range of cellular processes, especially for the immune response. Thus, we tried to evaluated how PB1 and their induced miRNAs affect the MAPK, NF-kB, and IFN-a signal pathways by western blot. BMDCs were transfect with PB1, miR375, miR181b, In-miR375, and In-miR181b for 48 h. Then cells were collected and washed with PBS three times for the next experiments. Western blot detection was performed as previously described by us. Mouse IkBa, P-IkBa, P38, P-P38, ERK, P-ERK, JUK, P-JUK, IRF-3, and IRF-7 were purchased from Abcam or Cell Signaling Technology and detected according to each manufacturer's protocol. Protein bands were visualized using the Super ECL Plus system. GAPDH was used as a loading control (Abcam).

### Statistical analyses

Data were evaluated by unpaired two-tailed Student's *t*-test using GraphPad Prism 5 (http://www.graphpad.com) (CSSN), with *p* < 0.05 considered to be statistically significant. The significance of the data was also determined by one-way ANOVA, followed by Tukey's multiple comparison tests. FACS data were analyzed by FlowJo software (FlowJo, China). All data are expressed as mean ± standard error of the mean.

## Author contributions

JL design and performed all the experiments, analyzed the data and drafted the manuscript, JX and CT developed the dendritic cells and performed flow cytometry analyses, KZ charged for the data analyzed, YZ and QY supervised the experiment and participated in the design. All the authors read and approved the final manuscript.

### Conflict of interest statement

The authors declare that the research was conducted in the absence of any commercial or financial relationships that could be construed as a potential conflict of interest.

## Supplementary material

Supplementary Image 1 **Identification and construction of pcDNA3.1-PB1, pcDNA3.1-PA, and pcDNA3.1-NP. (A)** Identification of pcDNA3.1-PB1 by digestion with *XhoI and KpnI* (M1: DL5000 DNA marker; 1: plasmid pcDNA3.1-PB1; 2: plasmid pcDNA3.1-PB1 digested with *XhoI and KpnI*). **(B)** Identification of pcDNA3.1-NP by digestion with *XhoI and HindIII* (M1: DL5000 DNA marker; 1: plasmid pcDNA3.1-NP; 2: plasmid pcDNA3.1-NP digested with *XhoI and HindIII*). **(C)** Identification of pcDNA3.1-PA by digestion with *XhoI and HindIII* (M1: DL5000 DNA marker; 1-3: plasmid pcDNA3.1-PA digested with *XhoI and HindIII*; 4: plasmid pcDNA3.1-PA).

Supplementary Image 2 **Identification and construction of pSilencer-miR375 and pSilencer-miR181b by digestion with *Bam*HI and *Hin*dIII. (A)** Identification of pSilencer-miR181b by digestion with *Bam*HI and *Hin*dIII (M1: DL5000 DNA marker; 1: plasmid pSilencer- miR181b; 2: pSilencer-miR181b digested with *Bam*HI and *Hin*dIII). **(B)** Identification of pSilencer-miR375 by digestion with *Bam*HI and *Hin*dIII (M1: DL5000 DNA marker; 1: pSilencer-miR375 digested with *Bam*HI and *Hin*dIII; 2: plasmid pSilencer-miR375).

Supplementary Table 1 **Primers used in amplified PB1, PA and NP**.

Supplementary Table 2 **qRT-PCR primers used for detecting miRNAs alteration**.

Supplementary Table 3 **qRT-PCR primers used for detecting target genes and viral segments**.

Supplementary Table 4 **Primers used in amplified miR375 and miR181b1**.

Supplementary Data Sheet 1 **Results of the qPCR analysis of the transfection efficient on BMDCs with plasmid pcDNA3.1-PA, pcDNA3.1-NP and pcDNA3.1-PB1**.
