# Supplementary figures and images for: H9N2 Avian Influenza Virus Protein PB1 Enhances the Immune Responses of Bone Marrow-Derived Dendritic Cells by Down-Regulating miR375
Source: Front Microbiol. 2017 Mar 22;8:287. doi: 10.3389/fmicb.2017.00287 (PMC5360757; doi:10.3389/fmicb.2017.00287)

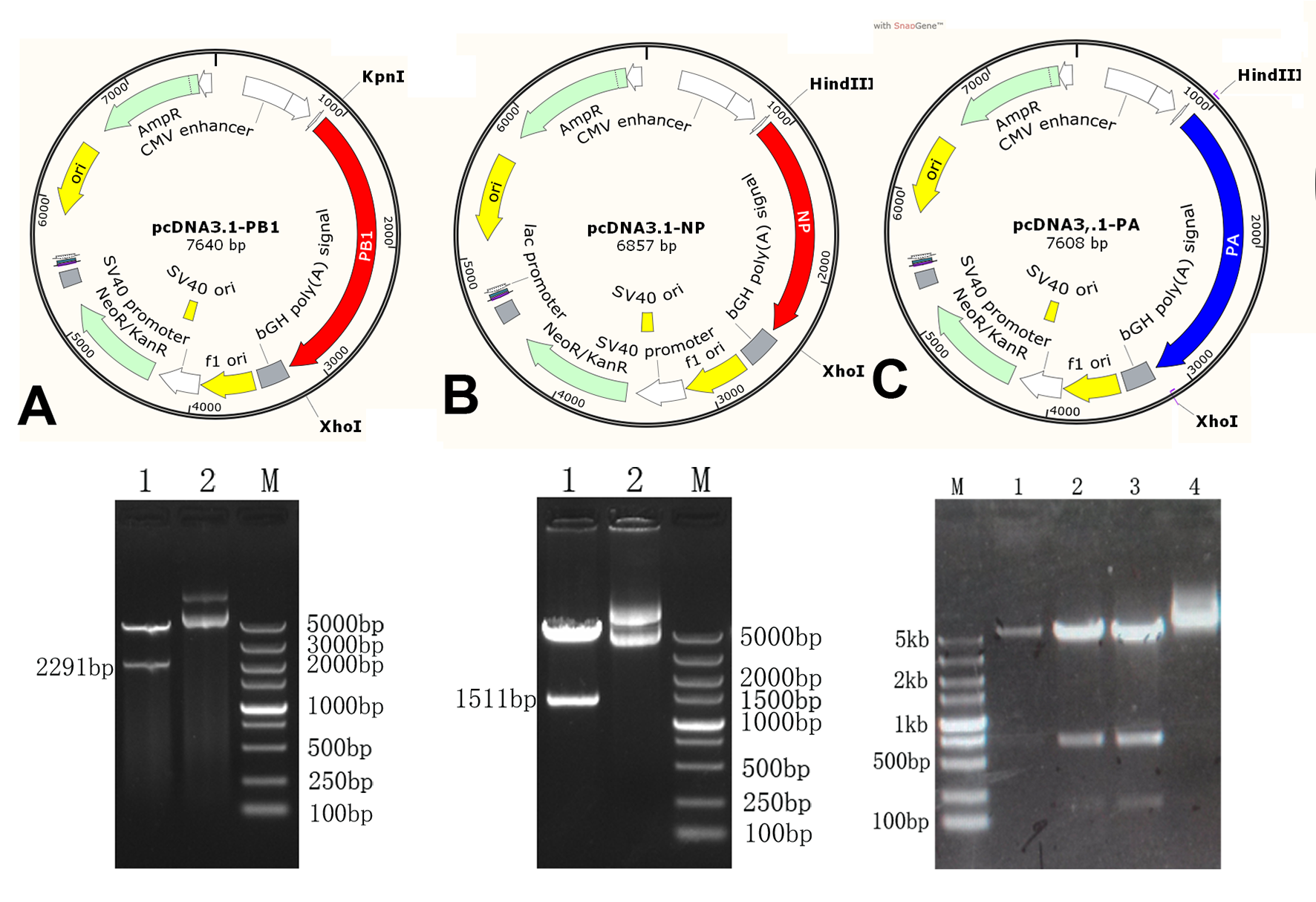

Supplement: Supplementary Image 1 — Identification and construction of pcDNA3.1-PB1, pcDNA3.1-PA, and pcDNA3.1-NP. (A) Identification of pcDNA3.1-PB1 by digestion with XhoI and KpnI (M1: DL5000 DNA marker; 1: plasmid pcDNA3.1-PB1; 2: plasmid pcDNA3.1-PB1 digested with XhoI and KpnI). (B) Identification of pcDNA3.1-NP by digestion with XhoI and HindIII (M1: DL5000 DNA marker; 1: plasmid pcDNA3.1-NP; 2: plasmid pcDNA3.1-NP digested with XhoI and HindIII). (C) Identification of pcDNA3.1-PA by digestion with XhoI and HindIII (M1: DL5000 DNA marker; 1-3: plasmid pcDNA3.1-PA digested with XhoI and HindIII; 4: plasmid pcDNA3.1-PA). [file Image1.TIF]

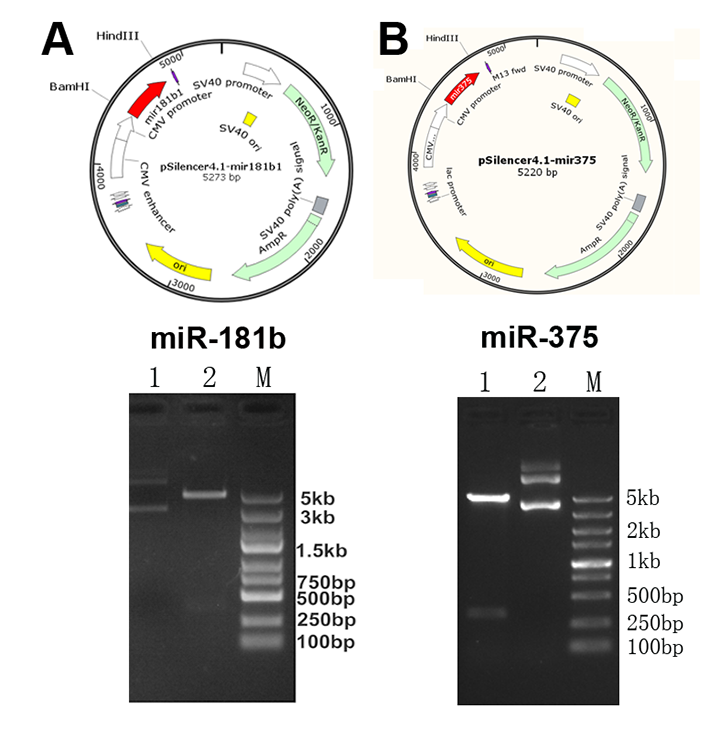

Supplement: Supplementary Image 2 — Identification and construction of pSilencer-miR375 and pSilencer-miR181b by digestion with BamHI and HindIII. (A) Identification of pSilencer-miR181b by digestion with BamHI and HindIII (M1: DL5000 DNA marker; 1: plasmid pSilencer- miR181b; 2: pSilencer-miR181b digested with BamHI and HindIII). (B) Identification of pSilencer-miR375 by digestion with BamHI and HindIII (M1: DL5000 DNA marker; 1: pSilencer-miR375 digested with BamHI and HindIII; 2: plasmid pSilencer-miR375). [file Image2.TIF]
