# Supplementary material for: H9N2 Avian Influenza Virus Protein PB1 Enhances the Immune Responses of Bone Marrow-Derived Dendritic Cells by Down-Regulating miR375
Source: Front Microbiol. 2017 Mar 22;8:287. doi: 10.3389/fmicb.2017.00287 (PMC5360757; doi:10.3389/fmicb.2017.00287)
Supplement: Supplementary Table 1 — Primers used in amplified PB1, PA and NP. [file Table1.DOC]

**Supplement table.1 Primers used in amplified PB1, PA and NP**

| **Gene** | **Sequence** | **Products** |
| --- | --- | --- |
| **PB-1 Sence** | **G*GGTACC*ATGGATGTCAATCCGAC** | **2291 bp** |
| **PB-1 Anti-sence** | **G*CTCGAG*CTATTTTTGCCGTCTG** |
| **PA Sence** | ***ACCCAAGCTGGCTAGC*ATGGAAGACTTTGTGCGACAATGCT** | **2151 bp** |
| **PA Anti-sence** | ***TGTTGTCCATAAGCTT*TCTCAGTGCATGTGTGAGGAAGGAG** |
| **NP Sence** | **G*AAGCTT*ATGGCGTCTCAAGGCAC** | **1511 bp** |
| **NP Anti-sence** | **G*CTCGAG*TCAATTGTCATACTCCTC** |
