# Supplementary material for: H9N2 Avian Influenza Virus Protein PB1 Enhances the Immune Responses of Bone Marrow-Derived Dendritic Cells by Down-Regulating miR375
Source: Front Microbiol. 2017 Mar 22;8:287. doi: 10.3389/fmicb.2017.00287 (PMC5360757; doi:10.3389/fmicb.2017.00287)
Supplement: Supplementary Table 2 — qRT-PCR primers used for detecting miRNAs alteration. [file Table2.DOC]

**Supplement table 2. qRT-PCR primers used for detecting miRNAs alteration**

| **MiRNA** | **Mibase number** | **Sence primer** |
| --- | --- | --- |
| **mmu-miR-155-5p** | **MIMAT0000165** | **GGGTTAATGCTAATTGTGATAGGGGT** |
| **mmu-mir-680-1** | **MI0004640** | **CCCGTAGACGACTGTACCCCC** |
| **mmu-miR-674-3p** | **MIMAT0003741** | **GGGCACAGCTCCCATCTCAGAACA** |
| **mmu-miR-222-5p** | **MIMAT0017061** | **GGGTCAGTAGCCAGTGTAGATCCT** |
| **mmu-miR-221-3p** | **MIMAT0000669** | **GGGAGCTACATTGTCTGCTGGG** |
| **mmu-miR-707** | **MIMAT0003497** | **CAGTCATGCCGCTTGCCTACG** |
| **mmu-mir-680-2** | **MI0004641** | **CGGGCATCTGCTGACATGGGGG** |
| **mmu-miR-22-3p** | **MIMAT0000531** | **GGAAGCTGCCAGTTGAAGAACTGT** |
| **mmu-miR-499-5p** | **MIMAT0003482** | **GGGGTTAAGACTTGCAGTGATGTTT** |
|  |  |  |
| **mmu-miR-375-3p** | **MIMAT0000739** | **TTTGTTCGTTCGGCTCGCGT** |
| **mmu-miR-29c-3p** | **MIMAT0000536** | **GGCGTAGCACCATTTGAAATCG** |
| **mmu-miR-146b-5p** | **MIMAT0003475** | **GGGGTGAGAACTGAATTCCATAGGCT** |
| **mmu-miR-687** | **MIMAT0003466** | **GGGCTATCCTGGAATGCAGCAATGA** |
| **mmu-miR-24-1-3p** | **MIMAT0000219** | **GGTGGCTCAGTTCAGCAGGAAC** |
| **mmu-miR-339-5p** | **MIMAT0000584** | **TCCCTGTCCTCCAGGAGCTCAC** |
| **mmu-miR-181b-5p** | **MIMAT0000673** | **AACATTCATTGCTGTCGGTGGGT** |
| **mmu-miR-679-3p** | **MIMAT0017248** | **GGAGCAAGGTCCTCCTCACAGTAG** |
