# Supplementary material for: H9N2 Avian Influenza Virus Protein PB1 Enhances the Immune Responses of Bone Marrow-Derived Dendritic Cells by Down-Regulating miR375
Source: Front Microbiol. 2017 Mar 22;8:287. doi: 10.3389/fmicb.2017.00287 (PMC5360757; doi:10.3389/fmicb.2017.00287)
Supplement: Supplementary Table 3 — qRT-PCR primers used for detecting target genes and viral segments. [file Table3.DOC]

**Supplement table 3. qRT-PCR primers used for detecting target genes and viral segments**

| **Gene name** | **Sence primer** | **Anti-Sence primer** |
| --- | --- | --- |
| PA | **CTCGCTCGTCAATGAAGTGG** | **AGCATGGAAGACTTTGTGCG** |
| NP | **AGAATCTCCCAATGCCGCTA** | **CGGAtGGCTCGAGTTTTCAG** |
| PB1 | **AGCGGGTATGCACAAACAGA** | **ATAAGTCTGGCGACCTTGGG** |
