# Supplementary material for: H9N2 Avian Influenza Virus Protein PB1 Enhances the Immune Responses of Bone Marrow-Derived Dendritic Cells by Down-Regulating miR375
Source: Front Microbiol. 2017 Mar 22;8:287. doi: 10.3389/fmicb.2017.00287 (PMC5360757; doi:10.3389/fmicb.2017.00287)
Supplement: Supplementary Table 4 — Primers used in amplified miR375 and miR181b1. [file Table4.DOC]

**Supplement table 4. Primers used in amplified miR375 and miR181b1**

| **Gene** | **Sequence** | **Products** |
| --- | --- | --- |
| **miR375 Sence** | **GGGATCCGAGAGCGGCGGCTAGC** | **482 bp** |
| **miR375 Anti-sence** | **GAAGCTTGAGTCTGAGGGCAGGGC** |  |
| **miR181b1 Sence** | **GCGGGATCCATTCAAATAAAAACCATC** | **312 bp** |
| **miR181b1 Anti-sence** | **GCGAAGCTTTTAGTGACTTCCTCACAG** |  |
